# Evaluation of the Relationship Between Echo Intensity and Young’s modulus of the Soleus Muscle Using Ultrasound Images After Ankle Fracture Surgery

**DOI:** 10.7759/cureus.69218

**Published:** 2024-09-11

**Authors:** Hayato Miyasaka, Bungo Ebihara, Takashi Fukaya, Hirotaka Mutsuzaki

**Affiliations:** 1 Department of Rehabilitation, Tsuchiura Kyodo General Hospital, Tsuchiura, JPN; 2 Graduate School of Health Sciences, Ibaraki Prefectural University of Health Sciences, Ami, JPN; 3 Department of Physical Therapy, Faculty of Health Sciences, Tsukuba International University, Tsuchiura, JPN; 4 Center for Medical Science, Ibaraki Prefectural University of Health Sciences, Ami, JPN; 5 Department of Orthopedic Surgery, Ibaraki Prefectural University of Health Sciences, Ami, JPN

**Keywords:** echo intensity, elastography, muscle quality, stiffness, young’s modulus

## Abstract

Objective: Although shear-wave elastography (SWE) can be used to assess muscle stiffness, SWE assessments are expensive. Echo intensity (EI) is an indicator of muscle quality and can potentially be used to assess muscle stiffness. This study aimed to determine the relationship between the EI and Young’s modulus of the soleus (SOL) muscle after ankle fracture surgery.

Methods: Eighteen participants who had undergone ankle fracture surgery were evaluated (mean age: 48.8 ± 20.6 years). Three months post-surgery, Young’s modulus and EI of the SOL muscle were measured using SWE and the combination of B-mode ultrasound and ImageJ software, respectively. EI and Young’s modulus measurements were obtained with the participant kneeling with knees bent 90°, upper body supported on a table, and ankles dorsiflexed 10°. The regions of interest used to measure EI and Young’s modulus were identical. The EI value corrected for the subcutaneous fat thickness was also calculated. Pearson’s correlation coefficients were calculated to examine the relationship of Young’s modulus with the uncorrected and corrected EI.

Results: Although the uncorrected EI was correlated with Young’s modulus of the SOL muscle (r = 0.567; p = 0.014), the corrected EI showed a stronger correlation (r = 0.637; p = 0.005). High intra-rater was also found reliability for the EI and Young’s modulus measurements of the SOL muscle in participants after ankle fracture surgery.

Conclusions: The EI and Young’s modulus of the SOL muscle were positively correlated. In particular, the corrected EI showed a stronger correlation with Young’s modulus than the uncorrected EI. Clinically, EI measurements may facilitate objective evaluation of muscle stiffness.

## Introduction

Ankle fractures are common fractures, accounting for 9% of all fractures [1,2]. These fractures can cause swelling, pain, and loss of flexibility [3]. After an ankle fracture, muscle stiffness limits the ankle's range of motion (ROM), adversely affecting daily life and return to work [3]. The postoperative management of ankle fractures often involves immobilisation in a cast or splint [4]. However, immobilisation and limited loading in the plantarflexed or neutral ankle positions can stiffen the soleus (SOL) muscle.

Shear-wave elastography (SWE) can be used to evaluate the stiffness of soft tissues such as muscles and tendons through measurements of Young’s modulus [5]. Young's modulus, a measure of tissue stiffness, is a value calculated from the external stress applied to an object and the strain caused by the stress. An increase in Young's modulus indicates tissue stiffness. However, SWE examinations are expensive and may not be practically applicable in many clinical and research institutions [6]. Thus, a less expensive method of evaluating muscle stiffness is required.

Echo intensity (EI) is an indicator of muscle quality and can be quantified using ultrasound B-mode images and the free software ImageJ [7]. EI measurements are obtained by setting a region of interest (ROI) in the B-mode image, which is then quantified on a 256-step scale, with 0 and 255 representing black and white, respectively [6]. In B-mode images, high levels of non-contracting tissues such as connective tissue and adipose tissue, which largely reflect ultrasound waves, are characterised by high muscle brightness [8].

Most of the existing studies on EI have focused on muscle strength [9], gait speed [10], and performance testing [11] in older adults. An increase in connective tissue is associated with muscle stiffness [12], and high muscle luminance may also result in high Young’s modulus values. In particular, ankle joint immobilisation and limitations of loading after ankle fracture may increase fibrosis of connective tissue and muscle and increase EI. Moreover, EI has been reported to be influenced by subcutaneous fat thickness; thus, EI corrected for subcutaneous fat thickness has been reported to correlate more strongly with lower-extremity function than uncorrected EI [9]. However, the relationship between the uncorrected and corrected EI and Young’s modulus after ankle fracture surgery remains unclear.

Thus, this study aimed to determine the relationship between EI and Young’s modulus of the SOL muscle after ankle fracture surgery. We hypothesised that EI and Young’s modulus are related and that the corrected EI is more strongly correlated with Young’s modulus than the uncorrected EI. The findings of this investigation may facilitate an objective assessment of muscle stiffness using EI measurements at various institutions.

## Materials and methods

Participants

The study was conducted between July 2022 and June 2024. The study population consisted of patients who had ankle fractures (malleolar, bimalleolar, or trimalleolar fractures) and were admitted to our hospital for open surgery and physiotherapy. The surgical treatment consisted of open reduction and internal fixation (ORIF). All participants were immobilised in a cast or splint for at least one week and used crutches for at least four weeks after surgery. Exclusion criteria included maximum ankle dorsiflexion ROM less than 10°, multiple fractures, open fractures, postoperative complications such as infection or deep vein thrombosis, history of neurological and orthopaedic disease, and transfer to another hospital. 

Measurements were performed three months after ORIF in 18 participants who met the inclusion criteria. All participants continued a program of training in mobility, muscle strength, and functional skills, such as walking and climbing stairs, at least once a week for three months. Data for the participants' age, sex, height, number of fractures [13,14], and Lauge-Hansen classification [15] were collected from the medical records. According to the Lauge-Hansen classification, fracture types are classified according to the injured limb position and the direction of external force. The participants’ weights were recorded using a digital scale and their body mass index values were calculated.

This study was approved by the institutional ethics committee and was conducted in compliance with the Declaration of Helsinki.

Measurement of EI and Young’s modulus of SOL muscle

All ultrasound examinations were performed using a 2-10  MHz linear transducer (Supersonic Imaging, Aix-en-Provence, France). The same physical therapist with eight years of experience in musculoskeletal ultrasound evaluated the EI using the B-mode and Young’s modulus using the SWE Opt penetration mode. All ultrasound images were recorded along the longitudinal axis of the muscle fibres. The room temperature was maintained at 25°C [16]. The participants were instructed to relax during the measurements.

**Figure 1:**
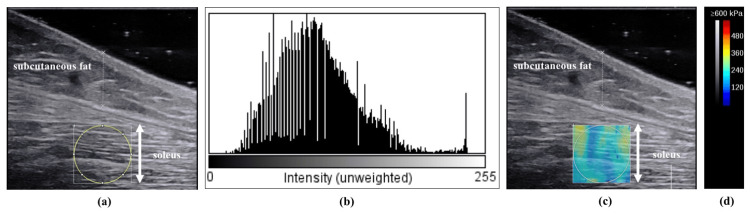
Typical examples of EI and Young’s modulus measurements. (a) B-mode images of the soleus muscle; (b) the value calculated by the Image J software indicates an average EI of 102.8 a.u.; (c) SWE images of the soleus muscle; (d) the colour scale. EI, echo intensity; SWE, shear-wave elastography.

Measurements of the EI and Young’s modulus of the SOL muscle were obtained with the participants kneeling with their knees bent 90°, upper body supported on a table, and ankle dorsiflexion of 10° [17]. The SOL measurements were obtained near the muscle-tendon transition region of the gastrocnemius [18]. For SOL measurements, the muscle-tendon transition zone was identified using B-mode horizontal axis images and marked on the skin with a black pen. In the B-mode measurements, the gain was set to 50%, and images were taken when bones and fascia were clearly visible. The dynamic range was 72 dB, and the focus depth was 2.0-4.0 cm in the middle of the SOL muscle. Young’s modulus was measured in the range of approximately 0-600 kPa. The ROI was a circle 10 mm in diameter set near the centre of the SOL muscle, and the same area was used for the EI and Young’s modulus measurements (Figure 1). The ROI is a partial region of interest in the image, and quantitative analysis is performed within this region. Sufficient amounts of gel were used to reduce the pressure on the skin. The EI was calculated as a 256-point value from 0 (black) to 255 (white) by converting the pixels of the saved B-mode image to 8-bit greyscale values using ImageJ software (National Institutes of Health, Bethesda, MD, USA) (Figure 1). Additionally, as outlined by Müller et al. [19], the EI value corrected for subcutaneous fat thickness was calculated using the following formula:

Corrected EI (a.u.) = uncorrected EI (a.u.)  − 5.0054 × (subcutaneous fat thickness)^2^ (cm) + 38.30836 × subcutaneous fat thickness (cm).

The subcutaneous fat thickness was measured to the nearest 0.1 cm using a digital measure inside the ultrasound device (Figure 1). To examine the reliability of the EI and Young’s modulus measurements, a second measurement was performed immediately after the first. Participants were told to maintain their regular activity level and refrain from impact activities two days before the measurements.

Measurement of ankle ROM

Ankle dorsiflexion and plantar flexion ROM measurements were obtained using a goniometer with a minimum value of 1° with the patient supine. The ankle dorsiflexion ROM was measured with the knee in the extended and flexed positions, and the plantar flexion ROM was measured with the knee in the flexed position. The participants dorsiflexed and plantarflexed their ankles as much as possible. During measurement, the lateral malleolus served as the fulcrum, the movement arm was parallel to the plantar surface of the foot, and the stationary arm was positioned relative to the long axis of the fibula.

Measurement of ankle strength

**Figure 2:**
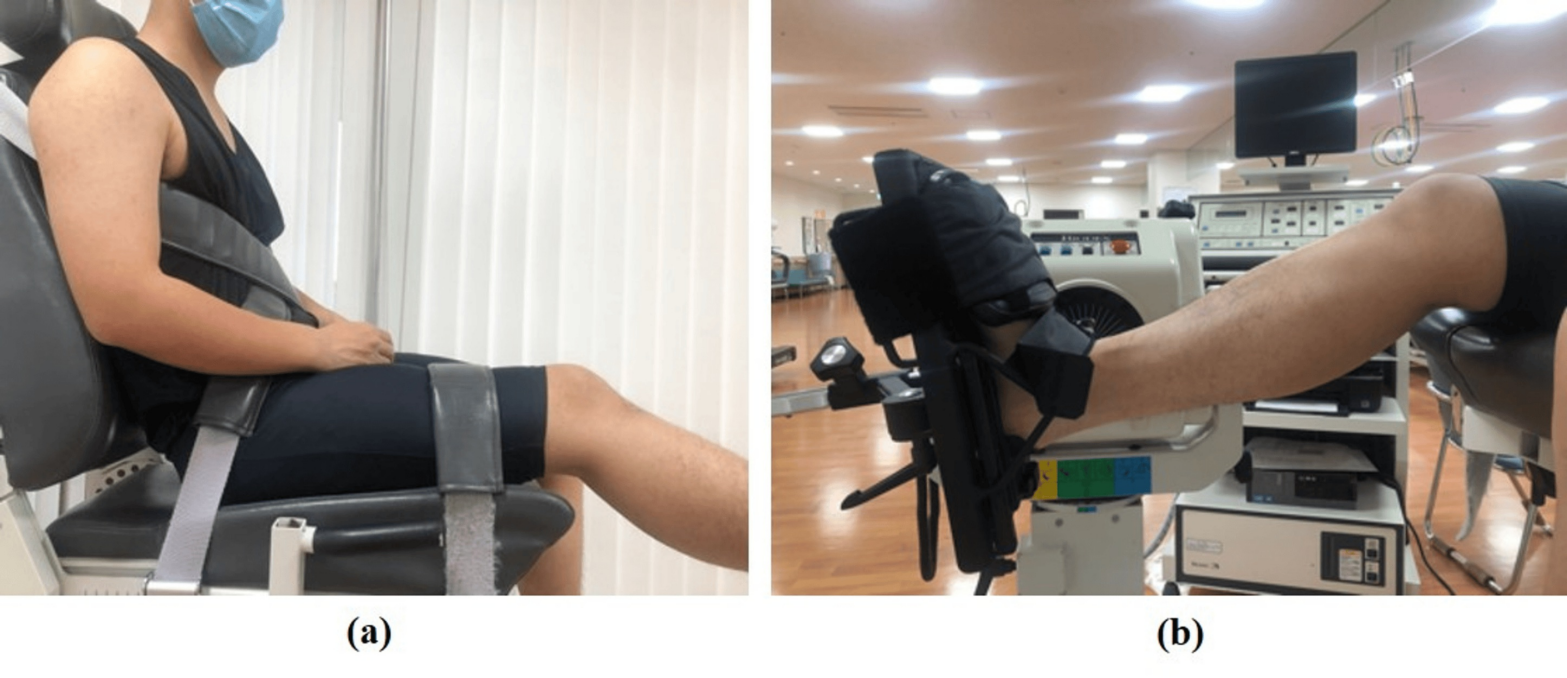
Illustration of the ankle strength measurement The measurements were obtained with the participants’ knees bent 30° while seated (a). Straps were used to stabilise the muscles of the ankle (b).

Ankle strength was measured using a Biodex 3 dynamometer (Biodex Medical Systems, Shirley, NY, USA) to evaluate the ankle plantar/dorsiflexion muscles. The measurements were obtained with the participants’ knees bent 30° while seated (Figure 2). Straps were used to stabilise the muscles of the lower trunk, thighs, and ankles (Figure 2). Ankle plantar/dorsiflexion measurements were performed bilaterally isokinetically (concentric/concentric), and two sets of five maximal dynamic repeats were performed at an angular velocity of 60°/s, separated by 30 s [18]. The participants were positioned such that their feet were parallel to the floor to prevent hamstring strain. Finally, the peak torque/body weight ratio was computed after the torque was measured at a minimum of 1 Nm.

Statistical analysis

G*power 3.1 (Heinrich Hein University, Germany) was used to calculate the sample size required (effect size = 0.61, α error = 0.05, power = 0.80), and the result was 18 [20]. The effect size of 0.61 was used based on a previous study that examined the correlation between EI and gastrocnemius muscle stiffness [6]. Therefore, 18 participants were included in this study. 

The Shapiro-Wilk test was used to evaluate the distribution of all data. The mean and standard deviation were obtained for data showing a regular distribution, whereas the median and interquartile range were noted for data with an irregular distribution. The intraclass correlation coefficient (ICC) and Bland-Altman plots were used to assess the reliability of the EI and Young’s modulus measurements. The average EI and Young’s modulus of the first and second measurements were used to calculate the ICC (1, 1) and for Bland-Altman analyses. To assess intra-rater reliability, the ICC (1, 1) was computed along with the standard error of the mean (SEM) [21], the 95% confidence interval of the minimum detectable change (MDC_95_) [22], and relative repeatability (RR) [23]. The formulas used to determine the SEM, MDC_95_, and RR are as follows: SEM = standard deviation × √(1 − ICC), MDC_95_ = 1.96 × SEM × √2, and RR = MDC_95_/mean, respectively. In addition, Pearson's correlation coefficients (r) were calculated to examine the relationship of EI with Young’s modulus and other measurement values.

Statistical significance was set at p < 0.05. All statistical analyses were performed using the Modified R commander version 2.8.1 (CRAN, Freeware).

## Results

Participant characteristics

The participant characteristics are summarised in Table 1. The mean age of the participants was 48.8 ± 20.6 years. The duration of ankle immobilisation and crutch use after ORIF were 14.0 (14.0-15.8) and 48.3 ± 11.8 days, respectively. The time from ORIF to measurement was 92.3 ± 5.8 days.

**Table 1:** Participants’ physical characteristics SER, supination-external rotation; PER, pronation external rotation; SA, supination-adduction. Values are presented as mean ± standard deviation orn/n.

Parameters	n = 18
Age (years)	48.8 ± 20.6
Sex	
Male	9
Female	9
Height (m)	1.61 ± 0.08
Weight (kg)	64.0 ± 14.1
Body mass index (kg/m^2^)	24.6 ± 4.4
Number of fractures	
I	9
II	6
III	3
Lauge–Hansen classification	
SER	9
PER	3
SA	6

Participants’ measured values

The measured values for the participants are listed in Table 2. The mean uncorrected EI, corrected EI, and Young’s modulus of the SOL muscle were 70.0 ± 19.4 a.u., 105.0 ± 18.2 a.u., and 58.0 ± 8.5 kPa, respectively.

**Table 2:** Participants’ measured values Values are presented as mean ± standard deviation.

Parameters	n = 18
Ankle range of motion (°)	
Dorsiflexion in the extended knee	14.2 ± 1.7
Dorsiflexion in the flexed knee	18.8 ± 2.3
Plantar flexion	59.4 ± 4.8
Ankle strength (Nm/kg)	
Plantar flexion	0.4 ± 0.1
Dorsiflexion	0.3 ± 0.1
Echo intensity (a.u.)	
Uncorrected	70.0 ± 19.4
Corrected	105.0 ± 18.2
Young’s modulus (kPa)	58.0 ± 8.5

Intra-rater reliability of the EI and Young’s modulus measurements of the SOL muscle

**Figure 3:**
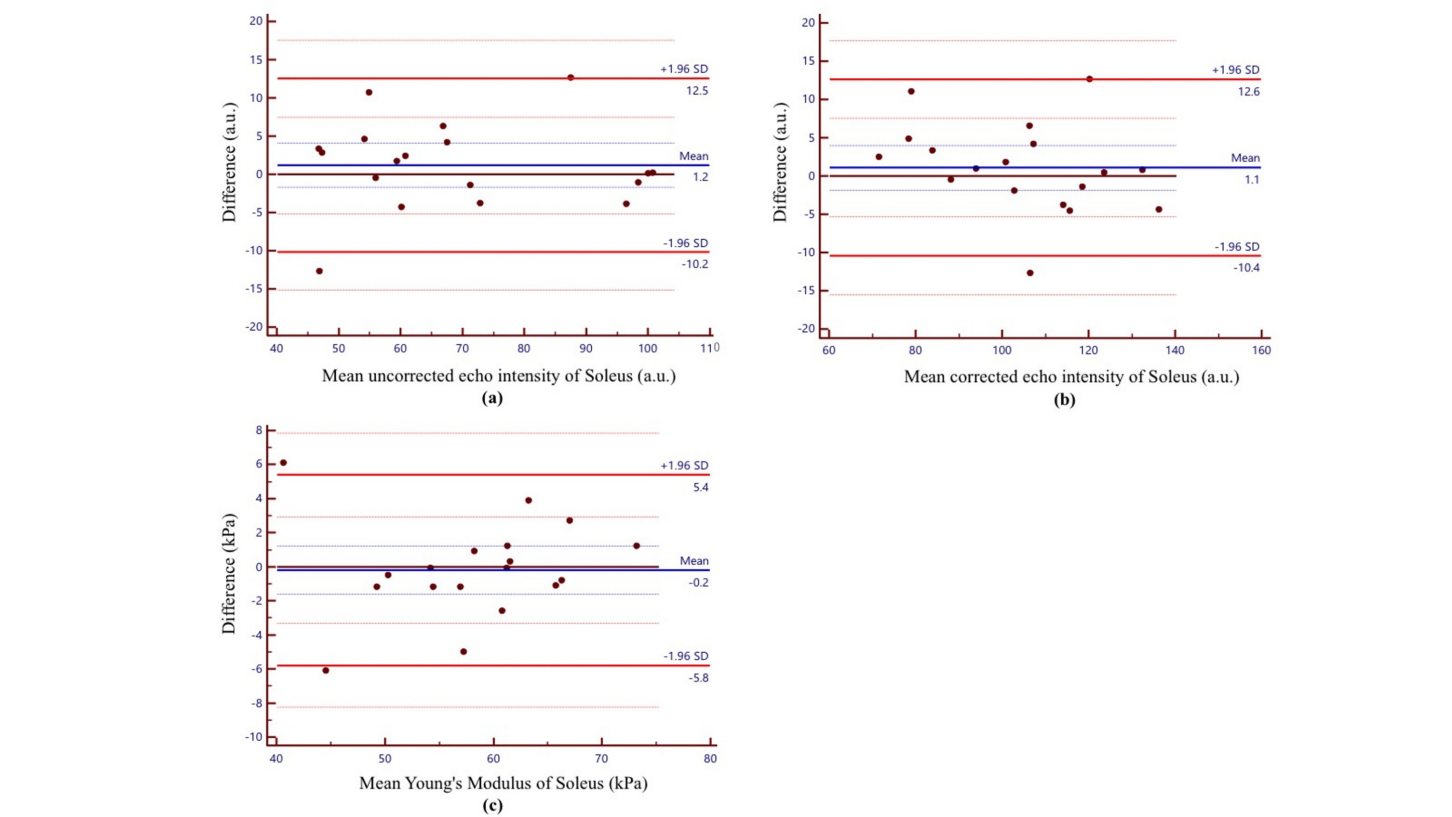
Bland-Altman plots of intra-rater reliability of the EI (a: uncorrected; b: corrected) and Young’s modulus measurements of the SOL muscle (c). The differences between the first and second EI and Young’s modulus measurements for the soleus muscle were plotted against the mean values for each participant. The blue line shows the mean of the differences, and the red line shows the limits of agreement ranging from -1.96 standard deviations to +1.96 standard deviations. The blue dotted line indicates the 95% confidence interval for the mean difference, and the red dotted line indicates the 95% confidence interval for the ±1.96 standard deviation. EI, echo intensity; SWE, shear-wave elastography.

The Bland-Altman plots of the intra-rater reliability values are shown in Figure 3. The mean differences in the uncorrected EI, corrected EI, and Young’s modulus of SOL muscle were 1.2 a.u., 1.0 a.u., and -0.2 kPa, respectively. The measurements showed no fixed or proportional bias, and the first and second measurements showed good agreement. The ICC, SEM, MDC_95_, and RR for the intra-rater reliability of the uncorrected EI, corrected EI, and Young’s modulus measurements are shown in Table 3. The ICC (1.1) values of the uncorrected EI, corrected EI, and Young’s modulus measurements were 0.96, 0.95, and 0.94, respectively; the SEM values were 4.09 a.u., 4.16 a.u., and 2.02 kPa, respectively; the MDC_95_ values were 11.3 a.u., 11.5 a.u., and 5.6 kPa, respectively; and the RR values were 0.16, 0.19, and 0.10, respectively.

**Table 3:** Intra-rater reliabilities of echo intensity and Young’s modulus measurements of the SOL muscle ICC, intraclass correlation coefficient; CI, confidence interval; SEM, standard error of the mean; MDC_95_, 95% confidence interval of the minimum detectable change; RR, relative repeatability; EI, echo intensity.

Measurement tissue	Test 1	Test 2	ICC	95% CI	SEM	MDC_95_	RR
Uncorrected EI (a.u.）	70.0	68.8	0.96	0.89-0.98	4.09	11.3	0.16
Corrected EI (a.u.）	105.0	104.0	0.95	0.88-0.98	4.16	11.5	0.19
Young’s modulus (kPa)	58.0	58.2	0.94	0.86-0.98	2.02	5.6	0.10

Correlation coefficients

The correlations between the EI and Young’s modulus of the SOL muscle and other measurement values are listed in Table 4. The uncorrected EI was correlated with Young’s modulus of the SOL muscle (r = 0.567; p = 0.014). The corrected EI was correlated with Young’s modulus of the SOL muscle (r = 0.637; p = 0.005), ankle dorsiflexion ROM in the flexed knee (r = -0.485; p = 0.042), and ankle plantar flexion strength (r = -0.589; p = 0.010). Young’s modulus of the SOL muscle was correlated with the ankle dorsiflexion ROM in the extended knee (r = -0.479; p = 0.044) and ankle dorsiflexion ROM in the flexed knee (r = -0.576; p = 0.012). The other measurement parameters were not significantly correlated (p > 0.05).

**Table 4:** Pearson’s correlation coefficients *Correlation was considered significant at p < 0.05. **Correlation was significant at p < 0.01.

Measurements	Uncorrected echo intensity		Corrected echo intensity		Young’s modulus	
	r	p-Value	r	p-Value	r	p-Value
Ankle range of motion						
Dorsiflexion in the extended knee	-0.188	0.456	-0.323	0.191	-0.479	0.044*
Dorsiflexion in the flexed knee	-0.224	0.373	-0.485	0.042*	-0.576	0.012*
Plantar flexion	0.164	0.515	-0.167	0.509	-0.102	0.688
Ankle strength						
Plantar flexion	-0.326	0.187	-0.589	0.010*	-0.067	0.792
Dorsiflexion	-0.111	0.662	-0.122	0.629	0.068	0.790
Young’s modulus	0.567	0.014*	0.637	0.005**	-	-

## Discussion

In this study, we aimed to determine the relationship between the EI and Young’s modulus of the SOL muscle after ankle fracture surgery. The findings of this study support our hypothesis that the EI and Young’s modulus of the SOL muscle are positively correlated. In particular, the corrected EI showed a stronger correlation with Young’s modulus, ankle dorsiflexion ROM, and ankle plantar flexion muscle strength than the uncorrected EI. However, Young’s modulus was more strongly correlated with the ankle dorsiflexion ROM than EI. 

Muscle quality has traditionally been associated with the strength and power per unit of muscle mass, and EI is used to estimate muscle quality [24,25]. Therefore, most previous studies on EI examined the relationship between EI and muscle strength and have suggested that EI reflects intramuscular fat content, with higher intramuscular fat content correlating with lower muscle quality [9,26,27]. However, EI also reflects connective and fibrous tissue content [8,28]. Honda et al. [12] reported that increased connective tissue is associated with muscle stiffness. Picelli et al. [29] also reported that patients with chronic stroke had a significantly higher gastrocnemius EI and significantly more limited passive ankle ROM than those with secondary progressive multiple sclerosis.

In this study, we found a positive correlation between EI and Young’s modulus of the SOL muscle in patients after ankle fracture surgery. Inactivity may affect ROM, EI, and muscle stiffness [30]. Inactivity of the ankle joint due to postoperative immobilisation and load limitation can increase the proportion of noncontractile tissues such as intramuscular fat and connective tissue, leading to increased muscle stiffness. The present study also found that the corrected EI correlated more strongly with Young’s modulus, ROM, and muscle strength than the uncorrected EI. These results suggest that correction for subcutaneous fat thickness may have a significant impact on the interpretation of muscle quality studies. Akima et al. [31] showed that subcutaneous fat thickness reduces muscle EI. Therefore, without correction, differences in muscle quality between participants with different subcutaneous fat levels may have been incorrectly assessed. Müller et al. [19] reported an equation that corrects EI for subcutaneous fat thickness. In several muscle quality studies, the corrected EI showed a stronger correlation with lower-extremity function than the uncorrected EI [26,32,33]. The results obtained after ankle fracture surgery in the present study also support the findings of these previous studies.

We also found high intra-rater reliability for the EI and Young’s modulus measurements of the SOL muscle in participants after ankle fracture surgery. Miyasaka et al. [18] reported high intra-rater reliability of Young’s modulus measurements of the SOL muscle in the knee flexion position, but only in healthy males. High reliability of muscle EI measurements has also been reported, but these studies were mostly conducted in healthy participants and athletes [6,34,35]. Moreover, the MDC_95_ was calculated in this study to provide values that reflected the true differences beyond the measurement error. The results showed that the MDC_95_ values for the uncorrected and corrected EI and Young’s modulus of the SOL muscle in the participants after ankle fracture surgery were 11.3 a.u., 11.5 a.u., and 5.6 kPa, respectively. Therefore, the EI and Young’s modulus of the SOL muscle must be greater than these values to reflect the effects of the intervention.

Clinically, the EI may serve as a tool for objectively evaluating muscle stiffness. This may be particularly useful for patients undergoing immobilisation and load limitations after ankle fracture surgery. Given that EI can be measured at a lower cost than SWE, EI measurements can be implemented at various institutions.

This study had several limitations. First, the results of this study may only partially reflect the quality of the SOL muscle. Second, fluid intake was not controlled before the EI measurement [36], and SOL activity was not monitored during the measurements. Therefore, the intracellular water content and muscle activity may have affected the EI and Young’s modulus measurements. Additional research is required to address these considerations.

## Conclusions

The EI and Young’s modulus of the SOL muscle were positively correlated after ankle fracture surgery. In particular, corrected EI showed better correlations with Young’s modulus, ankle dorsiflexion ROM, and ankle plantar flexion muscle strength than uncorrected EI. Thus, EI may serve as a low-cost objective tool for assessing muscle stiffness that can be implemented at various institutions.
